# Validation of the Lasher and Faulkender Anxiety about Aging Scale (AAS) for the Spanish Context

**DOI:** 10.3390/ijerph17124231

**Published:** 2020-06-13

**Authors:** Carolina Fernández-Jiménez, Joaquín F. Álvarez-Hernández, Darío Salguero-García, José M. Aguilar-Parra, Rubén Trigueros

**Affiliations:** 1 Department of Psychology, University of Granada, 18010 Granada, Spain; carolina@ugr.es; 2 Health Research Centre, Department of Psychology, Hum-878 Research Team, University of Almería, 04120 Almería, Spain; jalvarez@ual.es (J.F.Á.-H.); dariosalguero@ual.es (D.S.-G.); 3 Department of Language and Education, University of Antonio de Nebrija, 28015 Madrid, Spain

**Keywords:** anxiety, aging, factorial analysis, validation, attitudes toward aging, longevity

## Abstract

Increased longevity has led to concerns and fears among the population about the inexorable process of ageing. This process causes individuals to become more anxious about the physical and psychosocial changes caused by the passage of time. However, there are currently no scales in the Spanish context that analyse ageing. Therefore, the aim of the present study is to validate the Anxiety about Aging scale to the Spanish context. In the present study, 594 subjects between 25 and 64 years old participated. Both exploratory and confirmatory factor analyses were used. The results revealed that the factor structure of the questionnaire shows adequate psychometric properties, showing a four-factor factor structure.

## 1. Introduction

Technical and scientific progress in recent years has led to a global demographic change characterised by an ageing population. This increase in longevity has led to an interest in identifying the various physical and psychosocial factors that condition better adjustment to old age. Successful aging, characterized by high levels of well-being and personal satisfaction [1], would be determined to a large extent by the individual’s capacity to adapt to this final stage of life [2]. One of the factors that would mediate this adaptation process is anxiety in the face of aging [3,4,5,6]. However, at present there is no effective and valid instrument to assess anxiety about aging in the Spanish context, so this study proposes to adapt and validate the Anxiety about Aging scale [7].

Aging anxiety is a construct that differs from general anxiety in that it refers to the concerns and fears that we anticipate regarding the aging process traditionally associated with impairment, illness, and dependency [8,9]. Research has also indicated that anxiety about aging is an important mediating factor in attitudes and behavior toward older people. The results of these studies suggest that anxiety about aging is related to the presence of negative stereotypes about older people and aging [10,11,12,13]. The expression of these prejudiced attitudes and behaviours (ageism) appears to lead to discrimination and social exclusion [14], which has been shown to affect the physical and mental health of older people [15,16].

Research on aging anxiety has shown that this construct is related to different factors, with age and gender being the most widely studied. With respect to age, several studies have shown that young people have higher levels of anxiety about aging [17,18,19], probably due to fear of the unknown and a stereotypical misconception of this stage of life [19]. These findings are not conclusive, however, because in the few studies conducted with samples of middle-aged and older people, they showed more anxiety about old age than younger people [20,21]. These results could be explained by the influence of other conditions that occur in the transition to old age, such as retirement or deteriorating health, circumstances that can be experienced on a personal level as losses [17,22]. In short, the discrepancies found in the work carried out make it necessary to check the effect of age on the level of anxiety in old age in future studies.

Gender has also been shown to condition fear and concern about aging. Research findings have revealed that women and men experience ageing differently, and hence their fears of ageing are also different depending on the time of life [8]. However, while some studies indicate that men are more anxious about aging [20,23,24]. Other work indicates just the opposite, with women more fearful and concerned about aging, especially in terms of physical appearance [21,25,26,27].

The Anxiety about Aging scale (AAS) by Lasher and Faulkender [7] is one of the most widely used instruments for measuring anxiety in old age. This scale was developed by the authors to measure the concern and anticipation of the various losses that can occur physically, mentally and personally during the aging process. AAS captures four different aspects of anxiety in old age: physical, psychological, social and transpersonal. The physical dimension refers to perceived health status, physical changes associated with age, as well as concerns about sexuality and physical self-efficacy. The psychological dimension refers to the perceived level of control, dependency problems, self-esteem, life satisfaction and psychological disorders. The social dimension includes, among other aspects, social and economic losses, living conditions and perceived social support. Finally, the transpersonal dimension refers to coping with one’s own death, the search for meaning in past and present life events, as well as identity and relationship with the divine. These dimensions are expressed in three specific fears: fear of aging or the aging process itself, fear of being an older person, and fear or anxiety about older people. This tool was developed by the authors using a sample of 312 Americans between the ages of 25 and 74. The instrument initially had 84 items that were later reduced to 20 depending on the factorial solutions. Using a principal component analysis, with direct obliminal rotation with zero range, the authors identified four factors: Factor 1, Fear of Older People; Factor 2, Physical Appearance; Factor 3, Fear of Loss and Factor 4, Psychological Concerns, which explained 50.6% of the variance, obtaining an internal consistency by Cronbach’s alpha method of 0.78, 0.74, 0.71 and 0.69, respectively, and 0.82 for the full scale.

Several studies have corroborated the factorial structure found by the authors of the scale [18,27,28,29,30,31], although discrepancies have been found in the number and meaning of items according to age and gender when adapting the instrument to different contexts [18,32].

The aim of this study is to validate the Anxiety about Aging scale (AAS; [7]). For this purpose, two factor analyses (an exploratory and a confirmatory one) will be performed and reliability will be analyzed through the cronbach alpha index and temporal stability. In this way, it is expected that the results of the present study will show that the factorial structure of the questionnaire identifies three factors that would correspond to three types of fears: fear of aging or the aging process itself, fear of being an older person and fear or anxiety of older people.

## 2. Method

### 2.1. Participants Sample

The sample of participants belonged to the province of Granada, specifically workers from the University of Granada. There were 235 men and 359 women between the ages of 25 and 64 (M = 41.34; SD = 12.04). In addition, 82 men and 75 women participated (M = 42.17; SD = 7.12) to carry out the analysis of temporal stability. The sampling method was incidental non-probabilistic.

### 2.2. Measurements

Anxiety about aging. The Anxiety about Aging scale (AAS; [7]) was validated and adapted to measure the Anxiety about Aging scale. This questionnaire consists of 20 items distributed among four factors: fear of aging, fear of physical changes, fear of loss and fear of the elderly. The answers indicated by the participants were through a Likert type scale ranging from 1 (strongly disagree) to 5 (strongly agree).

### 2.3. Procedure

Initially, the questionnaire was translated from English to Spanish following the direct-inverse translation strategy [33]. This consisted of a group of 3 translators with more than 15 years of experience and training in psychology who translated the original questionnaire, in English, into Spanish. Later, the translated version, in Spanish, was translated into the original language. The quality of the translation was evaluated based on the degree of similarity with the first version. Finally, the final Spanish version obtained was reviewed by psychological experts specializing in anxiety and depression so that they could indicate whether the items obtained were well designed to measure the construct under study.

While the final version of the questionnaire was being obtained, several day centers in the province of Granada were contacted and asked for their collaboration. Once the definitive version of the questionnaire was obtained, the persons responsible for the day centers were explained, as well as the participants, the purpose and objectives of the study. The only requirement that was requested of them in order to participate in the study was the delivery of the signed and completed informed consent.

The questionnaires were completed individually, on paper at the day center, with a member of the research group present to explain any possible doubts that may arise. The participants took about 12 min to complete the questionnaire.

### 2.4. Data Analysis

To analyze the psychometric properties of the instrument. Initially, the factorial structure of the questionnaire was analysed to determine its validity, performing an exploratory factor analysis (EFA) and a confirmatory factor analysis (CFA). In addition, once the factorial structure of the questionnaire was analysed, the invariance of the scale according to gender was analysed, measuring the equivalence and comparing the equality of the estimated parameters between the different groups. This procedure is recommended by Kline [34] and Byrne [35], which consists of analysing the adjustment of the different models to which restrictions are added by comparing them with the base model (the configurable model in which no restrictions would be imposed). In this regard, a comparison was made between the unrestricted model and the measurement weights model, where the factorial loads were equal between the groups; the structural covariance model, the variances and covariances of the structural part of the model were equal between the groups; the measurement residue model, the variances and covariances of the residue were equal between the groups; the structural weight model, where the measurement weights are equally constrained; and the structural residuals model, where the structural residues are similar. Subsequently, the reliability of the instrument was tested through the analysis of internal consistency and temporal stability. Finally, the mean, standard deviation and bivariate correlations of the factors in the questionnaire were analyzed.

The IBM statistical package, SPSS 25 and AMOS 20, was used for the analyses.

Before analyzing the factor structure of the questionnaire, the Mardia coefficient was calculated to ensure the multivariate normality of the data obtained, which were considered to be robust [36]. A CFA was then performed. The procedure for fitting the maximum likelihood estimation procedure was used along with 20,000 bootstrapping. The lack of normality did not affect the estimators, so they are robust [37].

The following indices were considered in relation to the scale model through the CFA: the incremental indexes (CFI, Comparative Fit Index; IFI, Incremental Fit Index; TLI, Tucker Lewis Index) whose score must be above 0.95 [38], χ^2^/df whose score must be below 3 [39], SRMR (Standardized Root Mean Square Residual) whose score must be below 0.06 [40] and finally the RMSEA (Root Mean Square Error of Approximation) with its confidence interval (CI) whose score must be below 0.06 [41]. However, these adjustment rates should be interpreted with caution given the restriction they present [42].

## 3. Results

### 3.1. Exploratory Factorial Analysis

First, an EFA was carried out using the main components with the 20 elements that make up the scale (Table 1). The results showed four components with own values above 1, which explained 42.48%, 44.51%, 48.67% and 56.17% of the variance in the total score.

### 3.2. Confirmatory Factorial Analysis

**Figure 1:**
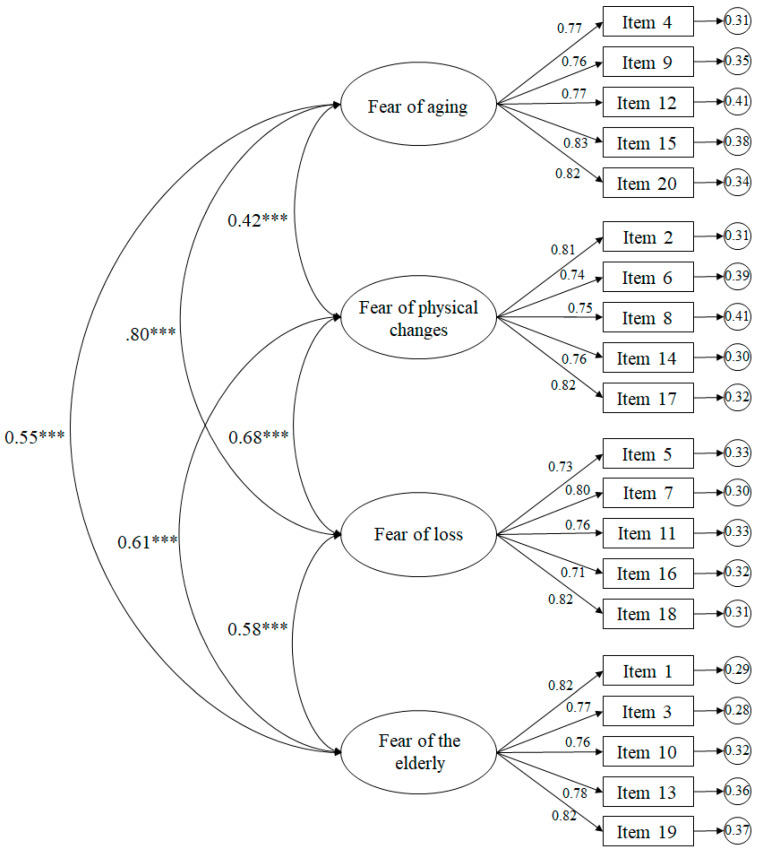
Confirmatory factor analysis (four factors). The ellipses represent the factors and the rectangles represent the different items. The residual variances are shown in the small circles. *** *p* < 0.001.

The adjustment rates for the CFA (Figure 1) were as follows: χ^2^ (164. N = 376) = 459.13, *p* < 0.001; χ^2^/df = 2.80; CFI = 0.95; TLI = 0.95; IFI = 0.95; RMSEA = 0.055 (CI 90% = 0.049–0.061); SRMR = 0.042. The correlations between factors were statistically significant (*p* < 0.001).

**Figure 2:**
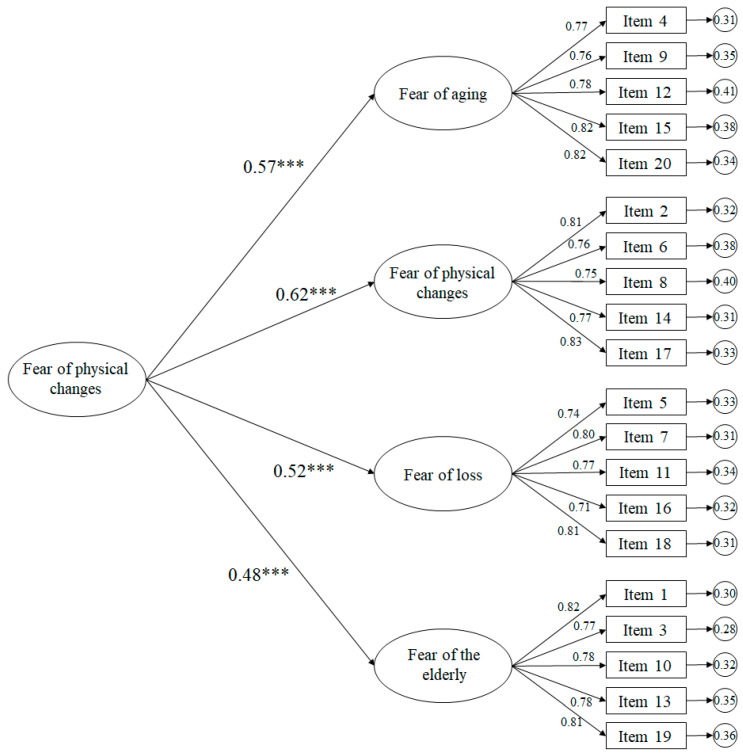
Confirmatory factor analysis (High-Order). The ellipses represent the factors and the rectangles represent the different items. The residual variances are shown in the small circles. *** *p* < 0.001.

In relation to the higher order model (Figure 2) the adjustment rates showed appropriate ones: χ^2^ (166. N = 376) = 535.79, *p* < 0.001; χ^2^/df = 3.23; CFI = 0.96; TLI = 0.96; IFI = 0.96; RMSEA = 0.061 (CI 90% = 0.056–0.067); SRMR = 0.047. There is a correlation between the higher order factor, called anxiety in old age, with respect to fear of aging of 0.57, fear of physical changes of 0.62, fear of loss of 0.52 and fear of the elderly of 0.48.

### 3.3. Gender Invariance Analysis

The nested models are analyzed with successive restrictions. The test of maximum probability ratio (χ^2^) was used to check these models. The difference (∆*χ*^2^) follows a *χ*^2^ distribution with degrees of freedom equal to the difference between the degrees of freedom (∆*df*). If this value is statistically significant in the second model, it means that the constraints specified in the more restrictive model are not maintained. Therefore, in the absence of significant differences between the first model and the second model it is a minimum criterion for accepting that the structure of the model is invariant with respect to gender [43,44]. In this regard, Table 2 shows that the fourth and third model has significant differences from the first model.

### 3.4. Descriptive Statistics, Correlation and Reliability Analysis

Table 3 shows Pearson’s mean, standard deviation and bivariate correlations. The correlations show a positive valence between the factors that are part of the questionnaire. In addition, it can be seen that the reliability analyses (Cronbach’s alpha and ICC; intra-class correlation coefficients) show a score above 0.80.

## 4. Discussion

Aging is a natural process characterized by maturative changes at the physical, psychological, social and emotional levels. However, there are currently no instruments in the Spanish context that assess how people deal with this process. Therefore, the aim of this study is to validate and adapt the AAS [7] to the context of the Spanish population by testing the psychometric properties of the instrument. The results of the study have shown that the AAS is an instrument that shows evidence of validity and reliability for measuring anxiety in old age in its four dimensions: fear of aging, fear of physical changes, fear of loss and fear or anxiety of the elderly. In this way, this instrument could help to better understand not only the fears and concerns that human beings have and are building up with respect to their own ageing process, but also about prejudices and behavioural attitudes towards the elderly and very elderly.

The results of this study, through both the EFA and the CFA, revealed that the factor structure of the AAS supports the three-factor model (fear of being an older person; fear of older people; and fear of the aging process itself). These results are in line with the results of the original scale, which reflected the same structure. In addition, the gender invariant analysis revealed that the factor structure of the questionnaire was gender invariant, so future studies could carry out comparative studies between men and women as the questionnaire is understood in a similar way by both populations. However, these results cannot be compared with the original scale, as the authors did not take this type of analysis into account. However, a study by Allan and Johnson [45] showed that the questionnaire was understood similarly by men and women in relation to their attitudes towards ageing. On the other hand, reliability analyses showed that Cronbach’s index scores were above 0.80 for all four factors, values above 0.70 being the limit set as acceptable [46]. These results were similar to those achieved in the original scale [7], as well as in several studies that have used the same instrument [31,47]. Finally, the results of temporal stability reflected that the scale is understood in a similar way by the whole population despite the passage of time, so it would be interesting to carry out future studies to analyse the perception of the population towards its own ageing at different moments in time.

We therefore understand that these results provide evidence in favour of the solidity of the structure of this measure and reveal that the adapted version manages to faithfully replicate the original theoretical structure. From now on, a short tool will be available, easy to apply for the administrators and easy to understand for the people evaluated in multiple contexts (e.g., health [48], social [45]). However, some limitations should be pointed out. Specifically, it must be demonstrated whether the invariance of the scale structure is met by age and socioeconomic level in order to corroborate the data obtained in this research. It will also be important that the scale could be interpreted on the basis of norm-referenced scores, such as percentile scores.

In conclusion, this version of the Ageing Anxiety Scale adapted to the Spanish context (Appendix A) has revealed satisfactory data fitting the underlying theoretical model and showing high internal consistency and validity. Given that anxiety about aging is related to the attitudes that we can generate towards the elderly and very old, ageist attitudes, attitudes that as Serrani-Azcurra [12] concludes “that negative ageist attitudes contribute to remove the feeling of fear referred to their future old age”, future use will be interesting.

## Figures and Tables

**Table 1:** Distribution of measurement weights.

Items	F1	F2	F3	F4
1				0.80
2		0.79		
3				0.79
4	0.81			
5			0.79	
6		0.78		
7			0.84	
8		0.83		
9	0.77			
10				0.76
11			0.77	
12	0.84			
13				0.81
14		0.80		
15	0.80			
16			0.75	
17		0.73		
18			0.82	
19				0.75
20	0.75			

Note. F1 = Fear of aging; F2 = Fear of physical changes; F3 = Fear of loss; F4 = Fear of the elderly.

**Table 2:** Gender invariance analysis.

**Four-Factor Model**										
**Models**	**χ^2^**	***df***	**χ^2^/*df***	**Δχ^2^**	**Δ*df***	**CFI**	**TLI**	**IFI**	**RMSEA (IC 90%)**	**SRMR**
Unconstrained	605.41	328	1.85	-	-	0.96	0.96	0.96	0.050 (0.044–0.060)	0.042
Measurement weights	619.22	344	1.80	21.31	16	0.96	0.96	0.96	0.053 (0.046–0.060)	0.044
Structural covariances	711.99	374	1.90	106.58	46	0.96	0.95	0.96	0.056 (0.049–0.061)	0.051
Measurement residuals	742.83	394	1.89	137.42 ***	66	0.95	0.95	0.95	0.060 (0.051–0.065)	0.054
**High-Order Model**										
**Models**	**χ^2^**	***df***	**χ^2^/*df***	**Δχ^2^**	**Δ*df***	**CFI**	**TLI**	**IFI**	**RMSEA (IC 90%)**	**SRMR**
Unconstrained	673.10	332	2.03	-	-	0.96	0.96	0.96	0.047 (0.042–0.055)	0.044
Measurement weights	685.71	348	2.14	18.61	16	0.96	0.96	0.96	0.051 (0.049–0.058)	0.049
Structural weights	784.15	368	2.13	82.13	36	0.95	0.95	0.95	0.055 (0.051–0.060)	0.051
Structural covariances	785.99	372	2.11	112.89 **	40	0.95	0.95	0.95	0.059 (0.053–0.068)	0.054
Structural residuals	788.86	376	2.10	115.56 ***	44	0.95	0.95	0.95	0.063 (0.056–0.069)	0.058
Measurement residuals	819.35	396	2.07	131.43 ***	64	0.95	0.94	0.95	0.065 (0.059–0.072)	0.061

*** *p* < 0.01; ** *p* < 0.001.

**Table 3:** Descriptive statistics, cronbach’s alpha, bivariate correlations and temporal stability analysis.

Factors	*M*	*SD*	α	1	2	3	4	ICC
1. Fear of aging	4.13	0.67	0.82		0.52 ***	0.48 ***	0.39 ***	0.82 (CI = 0.79–0.85)
2. Fear of physical changes	3.88	0.98	0.81			0.55 ***	0.43 ***	0.81 (CI = 0.78–0.86)
3. Fear of loss	3.57	0.87	0.80				0.32 ***	0.80 (CI = 0.77–0.84)
4. Fear of the elderly	3.64	0.93	0.86					0.85 (CI = 0.79–0.88)

*** *p* < 0.001.

## Appendix A

1. Disfruto estando con gente mayor.
2. Temo que cuando sea más mayor todos mis amigos se hayan muerto.
3. Me gusta visitar a mis familiares ancianos.
4. He mentido acerca de mi edad para parecer más joven.
5. Creo que será muy difícil para mí sentirme contento/a cuando sea anciano/a.
6. Para cuando sea mayor, lo que más me preocupa es mi salud.
7. Tendré mucho en que ocupar mi tiempo cuando sea mayor.
8. Me pongo nervioso/a cuando pienso que alguien tomará decisiones por mí cuando sea mayor.
9. Me molesta imaginarme siendo una persona mayor.
10. Disfruto hablando con gente mayor.
11. Cuando sea anciano/a creo que voy a sentirme bien con la vida.
12. Me preocupa el día en que al verme en el espejo me vea el cabello gris.
13. Me siento muy a gusto cuando estoy cerca de una persona mayor.
14. Me preocupa que la gente me ignore cuando sea anciano/a.
15. Imaginarme mayor me preocupa.
16. Creo que cuando sea anciano/a todavía podré hacer casi todas las cosas por mí mismo/a.
17. Me preocupa que la vida pierda sentido para mí cuando sea mayor.
18. Cuando sea mayor, confío en que me sentiré bien conmigo mismo/a.
19. Disfruto haciendo cosas por las personas mayores.
20. Cuando me miro al espejo me molesta observar que mi apariencia ha cambiado con la edad.
